# Biomechanical Correlates of Falls Risk in Gait Impaired Stroke Survivors

**DOI:** 10.3389/fphys.2022.833417

**Published:** 2022-03-07

**Authors:** Hanatsu Nagano, Catherine M. Said, Lisa James, William A. Sparrow, Rezaul Begg

**Affiliations:** ^1^Institute for Health and Sports (IHeS), Victoria University, Melbourne, VIC, Australia; ^2^Department of Physiotherapy, Melbourne School of Health Sciences, University of Melbourne, Melbourne, VIC, Australia; ^3^Department of Physiotherapy, Western Health, St. Albans, VIC, Australia; ^4^Australian Institute for Musculoskeletal Science, St. Albans, VIC, Australia; ^5^Department of Physiotherapy, Austin Health, Heidelberg, VIC, Australia

**Keywords:** stroke, falls prevention, gait retraining, tripping prevent, minimum foot clearance

## Abstract

Increased falls risk is prevalent among stroke survivors with gait impairments. Tripping is the leading cause of falls and it is highly associated with mid-swing Minimum Foot Clearance (MFC), when the foot’s vertical margin from the walking surface is minimal. The current study investigated MFC characteristics of post-stroke individuals (*n* = 40) and healthy senior controls (*n* = 21) during preferred speed treadmill walking, using an Optotrak 3D motion capture system to record foot-ground clearance. In addition to MFC, bi-lateral spatio-temporal gait parameters, including step length, step width and double support time, were obtained for the post-stroke group’s Unaffected and Affected limb and the control group’s Dominant and Non-dominant limbs. Statistical analysis of MFC included central tendency (mean, median), step-to-step variability (standard deviation and interquartile range) and distribution (skewness and kurtosis). In addition, the first percentile, that is the lowest 1% of MFC values (MFC 1%) were computed to identify very high-risk foot trajectory control. Spatio-temporal parameters were described using the mean and standard deviation with a 2 × 2 (Group × Limb) Multivariate Analysis of Variance applied to determine significant Group and Limb effects. Pearson’s correlations were used to reveal any interdependence between gait variables and MFC control. The main finding of the current research was that post-stroke group’s affected limb demonstrated lower MFC 1% with higher variability and lower kurtosis. Post-stroke gait was also characterised by shorter step length, larger step width and increased double support time. Gait retraining methods, such as using real-time biofeedback, would, therefore, be recommended for post-stroke individuals, allowing them to acquire optimum swing foot control and reduce their tripping risk by elevating the swing foot and improving step-to-step consistency in gait control.

## Introduction

A stroke is one of the most common and serious ageing-related health risks, with over 100 incidents documented in Australia every day (Australian Institute of Health and Welfare, 2020). Our primary concern in this report is the associated risk of falling within the year following a stroke because the likelihood of a fall in this population is 150% greater than in age- and gender-matched controls (Batchelor et al., 2012). Approximately 50% of post-stroke individuals residing at home are predicted to fall within 12 months (Mackintosh et al., 2005), with up to half evidencing multiple falls. An essential component of stroke patient care is, therefore, understanding stroke effects on gait control and, most importantly, *why* post-stroke gait impairments lead to a very high falls risk. Addressing this question is important in assisting physiotherapists to devise rehabilitation interventions that will not only improve stroke patients’ mobility but also make a significant contribution to decreasing their falls risk.

Across a range of conditions gait impairments commonly present as walking with shorter, wider steps, increased variability in step length and timing (Wang et al., 2019) and prolonged double support, when both feet are in contact with the ground (Hollman et al., 2011; Taniguchi et al., 2019). These gait characteristics are all typical of post-stroke individuals (Balasubramanian et al., 2009; Osada et al., 2021). Gait impairments can be viewed as reflecting clinically defined sensorimotor deficits (Callisaya et al., 2010; Taylor et al., 2012) but they also emerge as cautious gait adaptations to secure stability in response to a greater fear of falling (Young and Dingwell, 2012; Tsai and Lin, 2013; Bueno et al., 2019). It is, therefore, expected that in post-stroke individuals disrupted sensorimotor functions physically disturb healthy, vigorous walking (Jones and Adkins, 2015). An interesting question remains, however, as to why post-stroke individuals have such a high risk of falling when they *do* appear to employ cautious gait adaptations in an attempt to ensure their safety.

One approach to answering this question is recognising that tripping is the leading cause of falls, across all populations (Blake et al., 1988; Berg et al., 1997). Biomechanically, tripping can be characterised as unintentional swing foot contact with the walking surface, or an object on it, with sufficient momentum to induce forward balance loss (Smeesters et al., 2001; Nagano et al., 2011). The critical gait cycle event influencing tripping is Minimum Foot Clearance (MFC) at mid-swing, when the vertical margin between the lowest part of the foot and the walking surface is at its local minimum (Winter, 1991; Begg et al., 2007). In addition, the foot’s horizontal velocity is maximal at MFC, leading to a highly forceful impact in the event of obstacle contact (Winter, 1991). Swing phase foot trajectory control does not only maintain progression *via* displacement in the direction of travel, reflected in step length, but also modulates the vertical component to ensure safe and efficient foot-ground clearance. Ankle weakness (i.e., reduced dorsiflexors’ strength, plantar flexors’ contracture) due to a stroke can lead to reduced ankle dorsiflexion and, as a consequence, less foot-ground clearance (Blanton et al., 2002; Martin et al., 2015) and increased tripping risk. While increasing MFC height is, therefore, fundamental to preventing tripping, achieving consistent ground clearance, reflected in low MFC height variability, is also important because relatively few very low clearances will considerably elevate tripping risk (Begg et al., 2007).

Swing foot clearance is a complex, finely coordinated, sensorimotor function and, therefore, advanced, microanalysis is required to determine how MFC characteristics influence tripping (Begg et al., 2007). While traditional measures of central tendency and dispersion, such as the MFC mean and standard deviation provide a general description, Begg et al. (2007) developed methods to *predict* tripping risk by modelling the MFC height distribution of very large MFC samples from treadmill walking. Later, using the Begg et al. (2007) modelling, Nagano et al. (2011, 2020) applied bi-lateral MFC analysis to characterise asymmetrical swing foot control of the healthy senior population and post-stroke individuals.

The first question addressed in the current study was whether post-stroke people’s tripping related MFC characteristics could help to explain their high falls risk. The focus in this report, however, was the first percentile (1%) of the MFC distribution when tripping probability is highest. Increased MFC is fundamental to tripping prevention but the lowest segment of the dataset (i.e., the bottom 1%) may provide a more reliable foot-ground contact prediction than central tendency (i.e., mean or median). Most previous studies (Barrett et al., 2010) have characterised MFC height using the central tendency but it is reasonable to hypothesise that the best prediction of tripping is found in the infrequent very low swing foot clearances. In the current study, therefore, we hypothesised that post-stroke individuals would demonstrate lower 1% MFC with higher step-to-step variability indicating increased tripping risk.

Gait cycle parameters may, independently, reveal why post-stroke individuals are prone to falls despite cautious gait adaptations, reflected in variables, such as shorter and wider steps and prolonged double support (Young and Dingwell, 2012; Tsai and Lin, 2013; Bueno et al., 2019). Rinaldi and Monaco (2013), however, reported different *intralimb coordination patterns* for post-stroke individuals and healthy controls and proposed that walking mechanics can be considered a synthesis of multiple, interlinked, motor control processes, rather than separate systems. In this project, we extended these findings by identifying the correlation patterns between gait cycle variables, while conventional (mean ± standard deviation) gait cycle measures were maintained relatively constant using treadmill walking. We anticipated, for example, that correlation analysis would show whether cautious gait adaptations positively influence MFC; alternatively, it might be found that tripping risk is unaffected by gait improvements reflected in the timing and magnitude of traditional gait cycle events. This approach to stroke-affected gait analysis is innovative and a promising additional tool for understanding stroke effects on mobility. It may also inform physical rehabilitation procedures designed to correct gait defects and, most importantly, further our understanding of how rehabilitation procedures impact a patient’s falls risk. Correlations between MFC 1% and gait control variables, such as step width and length, and ankle dorsiflexion, for example, would suggest mobility treatment interventions that could promote safer swing phase ground clearance, particularly in the stroke patient’s more affected limb.

## Materials and Methods

### Participants

A total of 40 post-stroke individuals (Stroke) and 21 healthy senior controls (Control) participated in the study. The Stroke group (age 71 ± 12 years; height 1.69 ± 0.11 m; body mass 83.4 ± 17.6 kg) were all at least 6-month post-stroke. Participants who required gait aids in daily life, such as an ankle foot orthosis or walking stick, were included but the criteria for the current project also required the ability to walk at least 50 m without their assistive device while being tested. Post-stroke participants, therefore, had relatively higher mobility, confirmed by Stroke Rehabilitation Assessment of Movement (STREAM) scores of 87.4/100 for the lower extremity and 88.0/100 for mobility components. They had no other health conditions that prevented them from walking on a treadmill and, as indicated above, did not wear a foot orthosis or use an aid during testing. The Control group (age 74 ± 6 years; height 1.67 ± 0.09 m; body mass 71.6 ± 9.4 kg) were living independently, able to perform routine daily activities, free of any known cognitive, orthopaedic or neurological abnormalities and able to walk for at least 20 min continuously. Further inclusion criteria were below 12 s on a ‘timed up and go test’, a score of 20 or above on a visual contrast sensitivity test (‘Melbourne Edge Test’) and having not fallen within the previous 2 years. All post-stroke participants (Stroke) were volunteers and they provided informed consent using procedures approved by the Austin Health Human Research Ethics Committee. All healthy senior participants (Control) were volunteers recruited from the local community and the informed consent procedures mandated by the Victoria University Human Research Ethics Committee were applied.

### Testing Protocol

Gait testing was conducted on motor-driven treadmill at a pre-determined preferred speed (England and Granata, 2006). Stroke walked significantly slower (2.1 ± 1.0 km/h) than Control (3.6 ± 0.7 km/h; *t* = 5.906, *p* < 0.001). All participants were equipped with a safety harness, wore their own comfortable shoes and used the handrails to maintain stability (Wang et al., 2019). 3D kinematic position-time data were collected (100 Hz) using the Optotrak (NDI International, Waterloo, Ontario, Canada) with the two camera towers located bilaterally. Light-emitting diodes (LEDs) were utilised to model the foot kinematics. The key anatomical landmarks were heel, the proximal inferior surface of the shoe-outsole and the toe, the most anterior and superior toe part of a shoe. Treadmill walking continued for 10 min but for the Stroke group, testing was stopped when the supervising Physiotherapist or participants determined that a rest was required.

### Data Acquisition and Analysis

**Figure 1:**
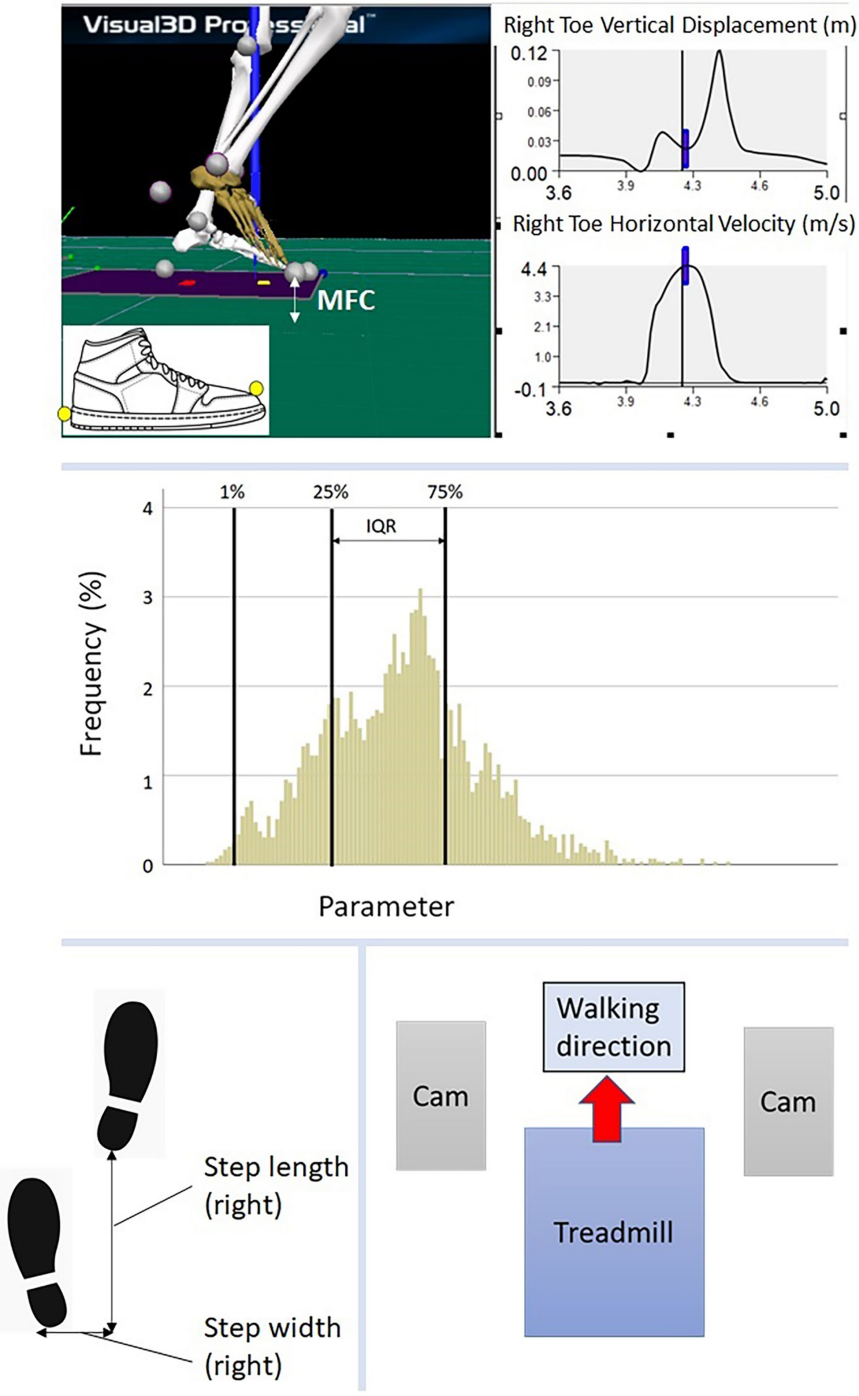
(top) Illustration of MFC, marker attachment at toe and heel; (middle) Histogram description, 1% = first percentile, 25% = 25th percentile, 75% = 75th percentile, IQR (interquartile range) = 75th-25th percentile; (bottom left) step length and step width; (bottom right) walking test environment, Cam = motion capture camera.

To identify heel contact and toe-off, we applied a foot velocity algorithm similar to that proposed by O’Connor et al. (2006). Examined parameters included step length, step width, double support and Minimum Foot Clearance (MFC). Step length and step width were defined, respectively, as anterior–posterior and medio-lateral displacement between heels at heel contact. Double support time was the temporal period from heel contact to contralateral toe-off. As illustrated in Figure 1 (top), MFC was defined as the mid-swing phase event where the vertical margin of the swing foot from the walking surface is at local minimum while moving at maximum velocity. In some cases, Stroke group demonstrated swing foot trajectories that did not show a conventionally defined MFC event and, in such circumstances, maximum swing foot horizontal velocity timing was used with vertical displacement at this time used to represent MFC height, using a previous procedure (Nagano et al., 2020).

**Table 1:** MFC characteristics of Post-stroke and Control groups.

	Stroke	Control	Group	Limb	Group × Limb
Mean	2.15 cm	2.35 cm	0.324	0.690	0.938
Median	2.10 cm	2.30 cm	0.406	0.680	0.933
1%	1.32 cm	1.93 cm	**<0.001**	0.500	0.958
SD	0.44 cm	0.32 cm	**0.003**	0.655	0.989
IQR	0.58 cm	0.45 cm	**0.025**	0.540	0.830
Skewness	0.39	0.89	**<0.001**	0.756	0.200
Kurtosis	0.47	0.97	0.149	0.603	**0.048**

*Significant effects (p < 0.05) indicated by bold. Main effects = Group, Limb; Interaction = Group × Limb*.

Raw 3D marker position/time coordinates were low-pass filtered using a fourth order zero-lag Butterworth Filter with a cutoff frequency of 6 Hz (Begg et al., 2007; Nagano et al., 2020). Spatio-temporal parameters were described using the mean and SD. MFC analysis followed the approach of Begg et al. (2007) using the central tendency (mean, median), step-to-step variability (SD and IQR) and MFC height distribution patterns (skewness and kurtosis). The MFC dataset was also separated at the first percentile (1%) as illustrated in Figure 1 (middle).

### Statistical Analysis

Multivariate Analysis of Variance (MANOVA) was applied with a 2 × 2 (Group × Limb) design for each spatio-temporal parameter’s mean and SD; while for MFC the median, the first (lowest) 1% of the height distribution, IQR, skewness and kurtosis were also obtained. Prior to MFC analysis, multivariate normality and homoscedasticity were confirmed by Mahalanobis distance and Homogeneity of variance (Levene’s Test of Equality of Error Variances) at the alpha level of 0.001. To identify hemiplegic stroke effects on gait variables the affected and unaffected limbs were compared. For healthy counterparts the non-dominant vs. dominant limb classification was employed (Seeley et al., 2008). Inspection of different MFC control processes between the groups was conducted by Pearson’s correlation analysis, applied to all dependent variables separately within each group to investigate their interaction with MFC control based on our previously used approaches (Nagano and Begg, 2020; Nagano et al., 2021). Significant effects were determined when computed *p*-values were less than 0.05.

## Results

### MFC

**Figure 2:**
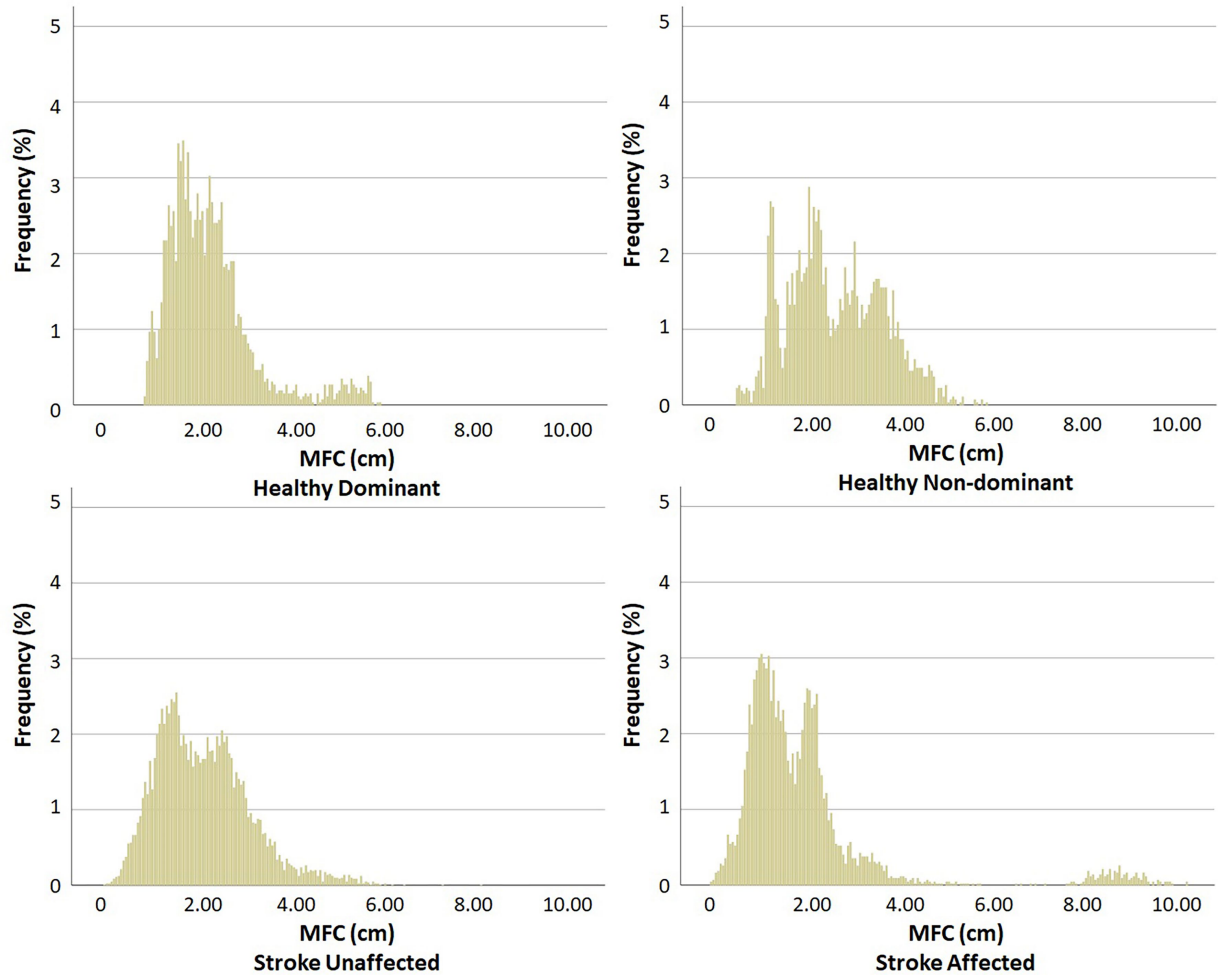
MFC histogram. Stronger vs. Weaker: Healthy (dominant vs. non-dominant) and Stroke (unaffected vs. affected).

Figure 2 displays the MFC histograms for Control and Stroke groups. Multivariate tests revealed an overall higher MFC in the Control group, as hypothesised (F_8, 111_ = 23.304, *p* < 0.001).

As shown in Table 1, MFC height was distinguished height was distinguished only in 1%, due to Stroke having 0.61 cm lower clearance (F_1, 118_ = 15.419, *p* < 0.001), as visualised in the lowest part of the dataset. Higher MFC variability was identified in the SD for Stroke by 0.121 cm (F_1, 118_ = 9.221, *p* = 0.003) and IQR by 0.135 cm (F_1, 118_ = 5.148, *p* = 0.025). Distribution pattern effects were revealed in lower skewness in Stroke (F_1, 118_ = 24.448, *p* < 0.001). In addition, a Group x Limb interaction was obtained for kurtosis (F_1, 118_ = 3.981, *p* = 0.048) with Stroke’s affected limb showing lower MFC kurtosis (0.211) compared to their unaffected side (0.727) and the Control dominant (0.537) and non-dominant limb (1.418).

### Spatio-Temporal Gait Parameters

**Figure 3:**
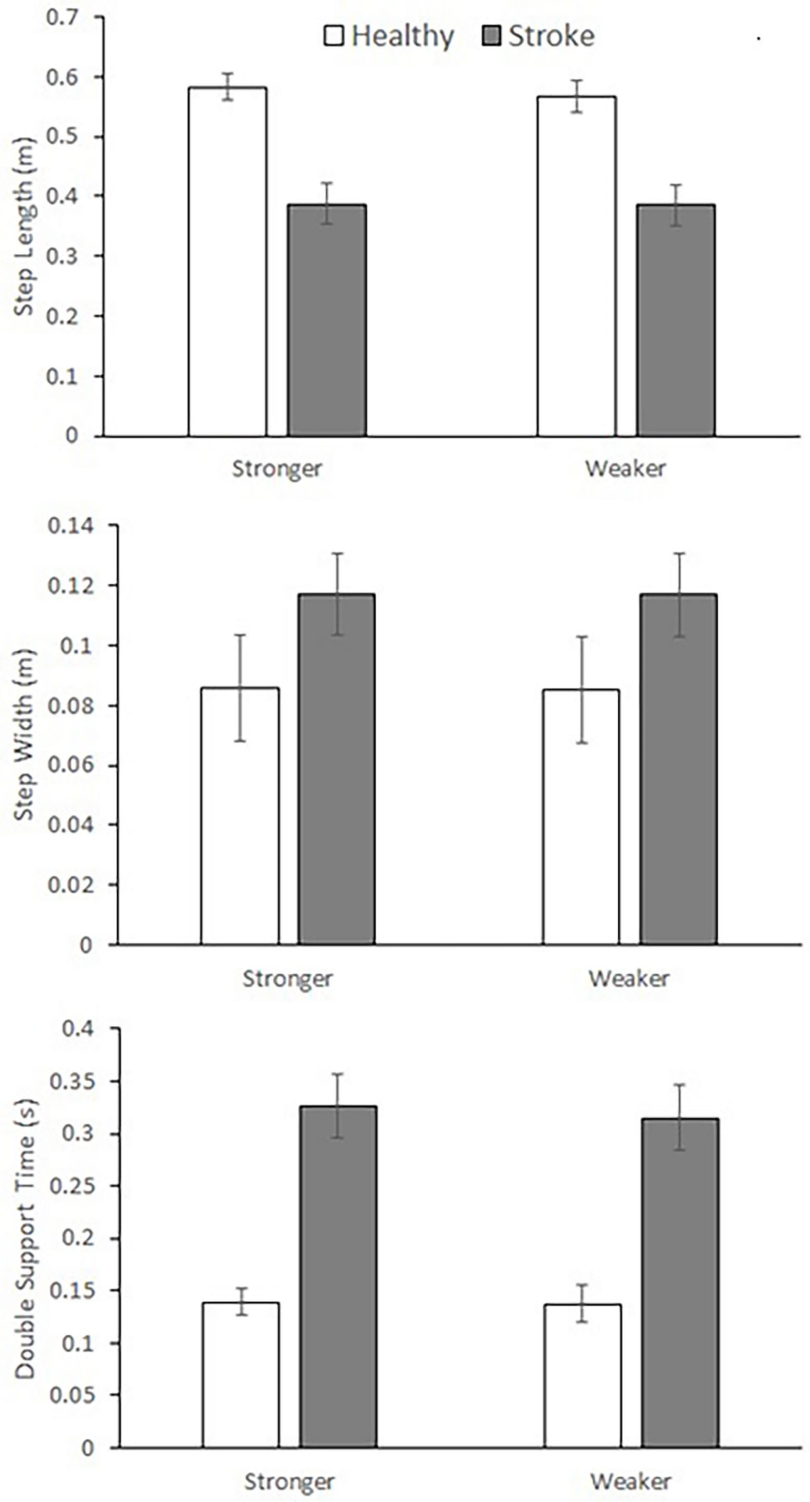
Spatio-temporal gait parameters (mean ± SD). (top) step length = group effects for mean and SD, (middle) step width = group effects for mean and SD, (bottom) double support time = group effects for mean and SD. Stronger vs. Weaker limb classification: healthy = dominant vs. non-dominant, Stroke = unaffected vs. affected.

Spatio-temporal parameters were clearly distinguished between the Control and Stroke for step length (F _7, 114_ = 11.603, *p* < 0.001), step width (F_7, 114_ = 8.168, *p* < 0.001) and double support time (F _7, 114_ = 20.485, *p* < 0.001), as in Figure 3.

As shown in the mean ± SD data (Figure 3), Stroke showed shorter (F_1, 120_ = 71.398, *p* < 0.001) and more variable (F_1, 120_ = 20.270, *p* < 0.001) step length, wider (F_1, 120_ = 10.726, *p* = 0.001) but less variable (F_1, 120_ = 22.642, *p* < 0.001) step width and greater (F_1, 118_ = 93.034, *p* < 0.001) but more variable double support time (F_1, 118_ = 15.130, *p* < 0.001).

### Correlations Between MFC Mechanics and Gait Control

Effects of stroke on gait coordination were determined using correlations analysis to reveal whether overall gait changes due to stroke would affect MFC control. Correlations patterns are reported below with respect to effects common to both groups, Stroke effects and Control effects.

#### Effects Common to Both Groups

Positive correlations were observed between MFC height and step-to-step MFC variability: Control (mean—SD: r = 0.682, *p* < 0.001; median—IQR: *r* = 0.736, *p* < 0.001) and Stroke (mean—SD: *r* = 624, *p* < 0.001; median—IQR: *r* = 0.436, *p* = 0.004), suggesting that increased MFC height tends to accompany higher step-to-step variability. MFC Skewness correlated negatively with height-related data for Control (mean *r* = −0.610, *p* = 0.003; median *r* = −0.622, *p* = 0.003; 1% *r* = −0.577, *p* = 0.006) and Stroke (mean *r* = −0.486, *p* < 0.001; median *r* = −0.509, *p* < 0.001; 1% *r* = −0.493, *p* = 0.001).

#### Stroke Effects

Step width correlated positively with mean (*r* = 0.320, *p* = 0.041) and median (*r* = 0.330, *p* = 0.035) MFC height but interestingly, positive correlations suggested that increased MFC 1% was accompanied with higher step width variability as in SD (*r* = 0.432, *p* = 0.005) and IQR (*r* = 0.445, *p* = 0.004). Step width kurtosis was negatively correlated with MFC variability for SD (*r* = −0.365, *p* = 0.019) and IQR (*r* = −0.334, *p* = 0.033), implying that variability of step width and MFC may be inter-coordinated. Increased *mean* step width was also associated with lower MFC skewness (*r* = −0.339, *p* = 0.030).

#### Control Effects

In contrast to the Stroke effects on MFC, Control showed negative correlations between mean step length and MFC variability: SD (*r* = −0.455, *p* = 0.038) and IQR (*r* = −0.433, *p* = 0.050), suggesting that longer steps are associated with less MFC variability.

## Discussion

Tripping is the leading cause of falls, particularly prevalent among the post-stroke population (Blake et al., 1988; Batchelor et al., 2012; Nagano et al., 2020). Previous studies have reported that higher tripping risk is reflected in lower and more variable MFC (Begg et al., 2007). Although the mean and median MFC did not distinguish the two groups’ MFC, 1% showed significantly lower MFC in the post-stroke group with larger step-to-step variability, possibly accounting for their previously reported 2.5 times higher falls risk compared to healthy age-matched controls (Mackintosh et al., 2005; Batchelor et al., 2012). Distribution patterns also highlighted the stroke-affected limb’s less kurtotic MFC characteristics, that, combined with greater SD and IQR, reflected more variable swing foot control, possibly due to impaired proprioception and rhythmicity (Warlop et al., 2018; Hamacher et al., 2019).

As expected from the report by Osada et al. (2021), spatio-temporal gait parameter analysis confirmed that the post-stroke group had critically impaired gait patterns compared to healthy seniors. This was indicated by typically shorter step length, larger step width and prolonged double support time (Taniguchi et al., 2019); similarly characterised by Balasubramanian et al. (2009). These gait adaptations were more clearly seen in slower gait (Wang et al., 2019), which could possibly explain why post-stroke individuals demonstrated overall less vigorous walking. The experimental protocol used in the current study closely followed Wang et al. (2019) and our findings were consistent for all spatio-temporal parameters except double support time variability. In their study, no differences were identified between the two groups while the current study revealed otherwise. This discrepancy may be attributable to differences in data volume with up to 10 min sampled at 100 Hz in our procedure compared to 20 s at 60 Hz by Wang et al. (2019). Furthermore, participants in the current study were 15–20 years older than the Wang et al. (2019) samples, suggesting that double support time variability may not be as pronounced in our relatively younger post-stroke individuals. Not only spatial gait control but also temporal aspects of motor function were impacted by stroke and rhythmic stepping exercises have been proposed as gait training to regain temporal gait coordination (Ghai and Ghai, 2019).

Among the examined spatio-temporal parameters, step width was highly interlinked with MFC control for the post-stroke individuals. Wider steps in the post-stroke group may have been an adaptation to compensate reduced dynamic balance by extending the base of support formed by the feet (Young et al., 2012; Onushko et al., 2019). Correlations suggested that wider steps could accompany higher MFC but importantly, this adaptation may not reduce tripping risk because increased MFC was observed only in central tendency (mean, median) but not in the first percentile (1%) in response to larger step width. Significantly lower MFC 1% among the post-stroke group was an important finding because it may explain the increased risk of tripping in this group. Attempts to increase MFC 1% are, therefore, arguably more important than *mean* or *median* for tripping prevention. According to correlations, however, elevated MFC 1% may accompany more variable step-to-step MFC control. This is a dilemma because both higher and consistent swing foot clearance should be required for prevention of tripping (Best and Begg, 2008) but the results suggest that improvement in one can negatively affect the other. For post-stroke individuals, gait training to reduce tripping risk should, therefore, challenge this undesirable MFC intralimb coordination by elevating MFC 1% while maintaining step-to-step consistency (Begg et al., 2014; Nagano et al., 2020).

**Figure 4:**
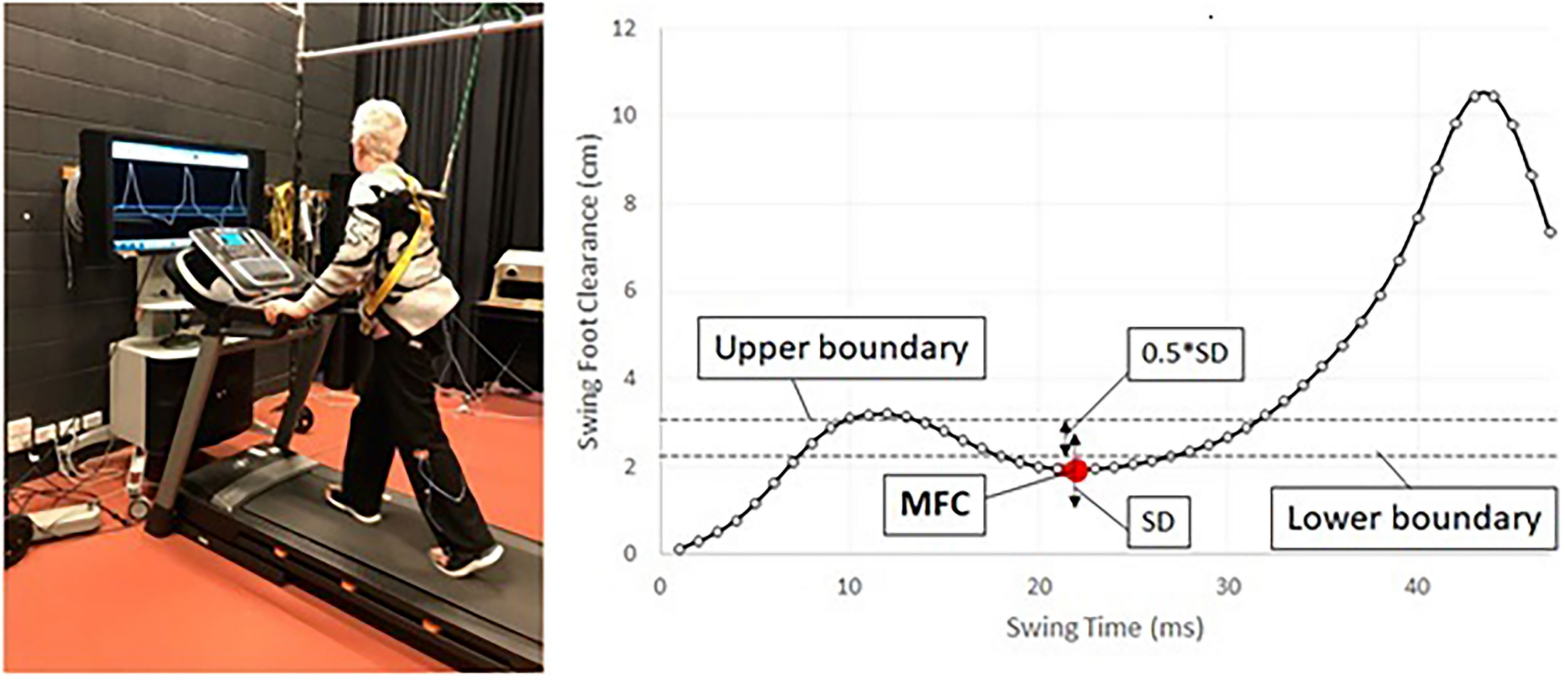
Protocol for biofeedback training for MFC. (left) Biofeedback training setup, (right) concept of MFC biofeedback training, red dot (MFC), targeting to control MFC within the band between upper/lower boundaries, target band = (mean + SD) ± (0.5*SD).

Biofeedback gait training for swing foot control can be suggested for this specific purpose (Begg et al., 2014), in which people can receive real-time visual feedback to control MFC within the target band, determined by individuals’ swing foot motions (Figure 4).

As visualised in Figure 4 (right), this training protocol is the attempt to control MFC within the narrow band to elevate swing foot clearance and improve consistency (Begg et al., 2014; Nagano et al., 2020). Other than MFC optimisation, various biofeedback training protocols have been proposed for the post-stroke population (Spencer et al., 2021). Auditory cueing (Mainka et al., 2018; Ghai and Ghai, 2019) and treadmill walking (Frenkel-Toledo et al., 2005) are the effective method to regain rhythmic stepping patterns (i.e., reduced step-to-step variability). Use of active exoskeletons is another approach for post-stroke people’s rehabilitation (Kaneko and Nakamura, 2010; Rodríquez-Fernández et al., 2021). While a number of intervention strategies are available, further validation work is necessary to confirm the most effective rehabilitation procedures for post-stroke individuals.

There were some limitations to the current study protocol. We permitted the use of treadmill handrails during testing, which may engender a more upright and symmetrical gait compared to walking freely. These results are consistent with Wang et al. (2019) allowing handrail use and reporting little asymmetry in gait patterns despite hemiplegic stroke. Compared to our previous studies that did not allow healthy older adults to use handrail support (Karaharju-Huisnan et al., 2001; Nagano et al., 2011, 2013), gait parameters were generally less asymmetrical. Without handrail support, however, treadmill walking is generally difficult for post-stroke individuals but in future studies overground protocols should be employed, to extend the findings to more typical everyday walking. Despite advantages of treadmill protocols for collecting a large volume of steady-state gait data, it is likely that some gait changes might be due to increased ‘fear of falling’ (Park and Yoo, 2014), particularly in the post-stroke population.

Multiple definitions of MFC were used for atypical swing foot clearance frequently observed in post-stroke individuals, in which maximum foot horizontal velocity during the swing phase was used as an alternative definition (Nagano et al., 2020). In addition, the participants wore their own preferred comfortable walking shoes which might also have caused minor differences in MFC definitions, however, each individual’s MFC was calculated relative to shoe’s ground reference to minimise this difference. In addition to large intra-subject variability in MFC control, the stroke group also demonstrated high inter-subject variability, visualised in the Figure 2 histograms. In future studies, post-stroke sub-populations could be investigated, based on, for example, severity of symptoms, time post-stroke and other health-related variables, such as body mass and cognitive function. Finally, in future MFC research, the focus could again be the lower end of the distribution (1% was selected in the current study) rather than central tendency because considerably greater tripping risk is associated with very low swing foot clearances. In addition, examining MFC characteristics for pre-determined low clearance thresholds, such as 1 mm (Killeen et al., 2017), is another potentially instructive analysis technique for investigating tripping risk in post stoke individuals.

## Data Availability Statement

The original contributions presented in the study are included in the article/supplementary material, further inquiries can be directed to the corresponding author.

## Ethics Statement

The studies involving human participants were reviewed and approved by the Austin Health Human Research Ethics Committee and Victoria University Human Research Ethics Committee. The patients/participants provided their written informed consent to participate in this study.

## Author Contributions

HN was responsible for data analysis and draft preparation. CS arranged the clinical research including recruitment of post-stroke individuals. LJ managed experimental procedures. WS edited the manuscript. RB coordinated the overall research project. All authors contributed to the article and approved the submitted version.

## Funding

The project was supported by the National Health and Medical Research Council (NHMRC) Project grant (approval number GNT1105800).

## Conflict of Interest

The authors declare that the research was conducted in the absence of any commercial or financial relationships that could be construed as a potential conflict of interest.

## Publisher’s Note

All claims expressed in this article are solely those of the authors and do not necessarily represent those of their affiliated organizations, or those of the publisher, the editors and the reviewers. Any product that may be evaluated in this article, or claim that may be made by its manufacturer, is not guaranteed or endorsed by the publisher.
